# Health-related quality of life in patients with type 1 diabetes mellitus in the different geographical regions of Brazil: data from the Brazilian Type 1 Diabetes Study Group

**DOI:** 10.1186/s13098-015-0081-9

**Published:** 2015-10-06

**Authors:** João Soares Felício, Ana Carolina Contente Braga de Souza, Camila Cavalcante Koury, João Felício Abrahão Neto, Karem Barbosa Miléo, Flávia Marques Santos, Ana Regina Bastos Motta, Denisson Dias Silva, Thaís Pontes Arbage, Carolina Tavares Carvalho, Hana Andrade de Rider Brito, Elizabeth Sumi Yamada, Roberta Arnoldi Cobas, Alessandra Matheus, Lucianne Tannus, Catia Cristina Sousa Palma, Leticia Japiassu, João Regis Ivar Carneiro, Melanie Rodacki, Lenita Zajdenverg, Neuza Braga Campos de Araújo, Marilena de Menezes Cordeiro, Jorge Luiz Luescher, Renata Szundy Berardo, Marcia Nery, Catarina Cani, Maria do Carmo Arruda Marques, Luiz Eduardo Calliari, Renata Maria de Noronha, Thais Della Manna, Roberta Savoldelli, Fernanda Garcia Penha, Milton Cesar Foss, Maria Cristina Foss-Freitas, Antonio Carlos Pires, Fernando Cesar Robles, Carlos Antonio Negrato, Maria de Fatima Guedes, Sergio Atala Dib, Patricia Dualib, Saulo Cavalcanti da Silva, Janice Sepúlveda, Emerson Sampaio, Rosangela Roginski Rea, Ana Cristina Ravazzani de Almeida Faria, Balduino Tschiedel, Suzana Lavigne, Gustavo Adolfo Cardozo, Mirela Azevedo, Luis Henrique Canani, Alessandra Teixeira Zucatti, Marisa Helena Cesar Coral, Daniela Aline Pereira, Luiz Antonio de Araujo, Hermelinda Cordeiro Pedrosa, Monica Tolentino, Flaviene Alves Prado, Nelson Rassi, Leticia Bretones de Araujo, Reine Marie Chaves Fonseca, Alexis Dourado Guedes, Odelisa Silva de Mattos, Manuel Faria, Rossana Azulay, Adriana Costa e Forti, Cristina Figueiredo Sampaio Façanha, Renan Montenegro Junior, Ana Paula Montenegro, Naira Horta Melo, Karla Freire Rezende, Alberto Ramos, Deborah Laredo Jezini, Marilia Brito Gomes, On behalf of the Brazilian Type 1 Diabetes Study Group (BrazDiab1SG)

**Affiliations:** Endocrinology Division, University Hospital João de Barros Barreto, Federal University of Pará, Mundurucus Street, 4487, Guamá, Belém, Pará 66073-000 Brazil; Diabetes Unit, Department of Internal Medicine, State University of Rio de Janeiro, Rio De Janeiro, Brazil; Federal University Hospital of Rio de Janeiro, Rio De Janeiro, Brazil; General Hospital of Bonsucesso, Rio De Janeiro, Brazil; University Hospital Clementino Fraga Filho, Children Institute Martagão Teixeira, Rio De Janeiro, Brazil; Diabetes Unit, University Hospital of São Paulo, São Paulo, Brazil; Pediatric Unit of Endocrinology, Santa Casa Hospital, São Paulo, Brazil; Children Institute of Endocrinology, University Hospital of São Paulo, São Paulo, Brazil; Ribeirão Preto Medical School of São Paulo University, Ribeirão Preto, Brazil; Department of Internal Medicine, Medical School, State University of São José do Rio Preto, São José Do Rio Preto, Brazil; Bauru’s Diabetics Association, Bauru, São Paulo Brazil; Diabetes Unit, Federal University of São Paulo State, São Paulo, Brazil; Endocrinology Unit, Hospital of Santa Casa of Belo Horizonte, Belo Horizonte, Minas Gerais Brazil; Diabetes Unit, State University Hospital of Londrina, Paraná, Brazil; Clinical Hospital of the Federal University of Paraná, Paraná, Brazil; Institute of Diabetic Children, Porto Alegre, Rio Grande do Sul Brazil; Clinical Hospital of Porto Alegre, Porto Alegre, Rio Grande do Sul Brazil; University Hospital of Santa Catarina, Florianópolis, Santa Catarina Brazil; Endocrinology and Diabetes Institute of Joinville, Joinville, Santa Catarina Brazil; Regional Hospital of Taguatinga, Brasília, Brazil; General Hospital of Goiânia, Goiânia, Brazil; Diabetes and Endocrinology Center of Bahia, Salvador, Bahia Brazil; Federal University of Maranhão, São Luís, Maranhão Brazil; Diabetes and Hypertension Center of Ceará, Fortaleza, Ceará Brazil; Federal University of Ceará, Fortaleza, Ceará Brazil; Federal University of Sergipe, São Cristóvão, Sergipe Brazil; Federal University Hospital of Campina Grande, Campina Grande, Paraíba Brazil; Getúlio Vargas University Hospital of Amazonas, Adriano Jorge Hospital, Manaus, Amazonas Brazil

**Keywords:** Type 1 diabetes mellitus, Health-related quality of life, EuroQol

## Abstract

**Background:**

In type 1 diabetes mellitus (T1DM) management, enhancing health-related quality of life (HRQoL) is as important as good metabolic control and prevention of secondary complications. This study aims to evaluate possible regional differences in HRQoL, demographic features and clinical characteristics of patients with T1DM in Brazil, a country of continental proportions, as well as investigate which variables could influence the HRQoL of these individuals and contribute to these regional disparities.

**Methods:**

This was a retrospective, cross-sectional, multicenter study performed by the *Brazilian Type 1 Diabetes Study Group* (BrazDiab1SG), by analyzing EuroQol scores from 3005 participants with T1DM, in 28 public clinics, among all geographical regions of Brazil. Data on demography, economic status, chronic complications, glycemic control and lipid profile were also collected.

**Results:**

We have found that the North-Northeast region presents a higher index in the assessment of the overall health status (EQ-VAS) compared to the Southeast (74.6 ± 30 and 70.4 ± 19, respectively; p < 0.05). In addition, North-Northeast presented a lower frequency of self-reported anxiety-depression compared to all regions of the country (North-Northeast: 1.53 ± 0.6; Southeast: 1.65 ± 0.7; South: 1.72 ± 0.7; Midwest: 1.67 ± 0.7; p < 0.05). These findings could not be entirely explained by the HbA1c levels or the other variables examined.

**Conclusions:**

Our study points to the existence of additional factors not yet evaluated that could be determinant in the HRQoL of people with T1DM and contribute to these regional disparities.

## Background

Type 1 diabetes mellitus (T1DM) affects the psychological and emotional well-being of patients and their families [1] and, in diabetes management, enhancing health-related quality of life (HRQoL) is as important as good metabolic control and prevention of secondary complications [2]. However, studies examining the HRQoL of people with T1DM are still limited [3]. This is the first multicenter population-based study on this topic conducted in the Southern hemisphere.

When studying this aspect, it is essential to first establish the conceptual difference between the terms quality of life (QoL), HRQoL, quality of health and health status. QoL is a multifaceted and highly subjective concept, and has been defined as “how good or bad a person feels their life to be” [4, 5]. The most suitable instruments to assess QoL are World Health Organization Quality of Life (WHOQOL), diabetes quality of life (DQOL) and audit of diabetes-dependent quality of life (ADDQoL) [4]. In contrast, HRQoL, quality of health and health status are more specific terms, and refer to how people feel about their physical and mental health [6]. HRQoL is more accurately measured by Short-Form 36 (SF-36) and EuroQoL 5-dimension (EQ-5D) [4].

With continental proportions, Brazil is the fifth largest country in the world, and covers a total area of over 8 million Km^2^. According to the latest population census, conducted in 2010 by the Brazilian Institute of Geography and Statistics (IBGE), Brazil has an estimated population of 190 million people, resulting in a demographic density of 22.5 inhabitants/km^2^ [7]. The Southeast region presents the highest population density, with 42 % of the Brazilian residents, followed by Northeast (27.7 %), South (14.3 %), North (8.5 %) and Midwest (7.5 %) [8].

Brazil is marked by its regional disparities, mainly centered on North and Northeast’s relative backwardness and predominantly unfavorable indicators [9]. According to IBGE’s survey, in 2013, Northeast and North presented the lowest urbanization rates (73.3 and 74.6 %, respectively) and the highest infant mortality rates (19.4 and 19.2 deaths per 1000 live births, respectively). Moreover, when compared to the other geographical regions, the North-Northeast region showed worse basic sanitation and health care provision indicators, higher fecundity and illiteracy rates, and lower asset ownership and percentage of people in formal work [7, 8].

In order to explore the possible impact of the noticeable regional heterogeneity of Brazil on health care and HRQoL in a selected group of people, the *Brazilian Type 1 Diabetes Study Group* (BrazDiab1SG) performed a survey that analyzed the demographic, clinical, laboratorial and economic data of 3591 patients with T1DM who received medical care at public clinics among all five geographical regions of Brazil. The purpose of the present study was to evaluate possible regional differences in HRQoL of patients with T1DM in Brazil, as well as investigate which variables could influence the HRQoL of these individuals and contribute to these regional disparities.

## Methods

### Study design

The BrazDiab1SG performed a retrospective, cross-sectional and multicenter study on people with T1DM between December 2008 and December 2010, in 28 public clinics of the secondary and tertiary care level, located in 20 cities (population greater than 100,000), in the five Brazilian geographical regions (North, Northeast, Southeast, South and Midwest). For statistical purposes, North and Northeast were grouped.

### Patients

All patients received health care from the National Brazilian Health Care System (NBHCS), and were treated by an endocrinologist in secondary or tertiary care settings. Were included on this study patients diagnosed with T1DM by a physician (based on a typical clinical presentation as well as the need of using insulin continuously since the diagnosis), with follow-up time in each center greater than or equal to 6 months, and older than 10 years old. Exclusion criteria were pregnancy or lactation (excluded by self-report), and history of acute infectious processes or diabetic ketoacidosis in the three months prior to assessment.

### Clinical and laboratorial data

Data of interest for the analysis of HRQoL were obtained from questionnaires self-completed by the patients, in addition to records obtained from medical charts. Trained physicians interviewed and examined all patients according to a standardized protocol, assessing current age, age at diagnosis of T1DM, duration of diabetes (years), monthly family income in minimum wages, economic class, physical activity, height (m), weight (kg), blood pressure (mmHg), insulin therapy regimen, diabetes-related comorbidities and smoking status (defined as smoking more than one cigarette per day at the time of the interview). Body mass index (BMI) (kg/m^2^) was determined by dividing the weight (kg) by height (m) squared. Practice of physical activity was addressed by a questionnaire that considered (1) only on weekends, (2) two to three times per week, (3) three to five times per week, (4) more than five times per week, and (5) no physical activity. For statistical analysis, we considered only whether patients practiced physical activity or not.

The economic status was defined according to the Brazilian Economic Classification Criteria [10], which considers asset ownership, access to public services and education level (categorized as illiterate/incomplete primary education, complete primary education/incomplete secondary education, complete secondary education/incomplete high school, complete high school/some college, or complete college education). For this analysis, the economic classes considered were high (A), medium (B1–B2), low (C1–C2) and very low (D–E).

The levels of glycated hemoglobin (HbA1c), fasting plasma glucose (FPG), total cholesterol, low-density lipoprotein (LDL) cholesterol, high-density lipoprotein (HDL) cholesterol, and triglycerides measured during the last clinical visit were obtained from the participants’ medical records. HbA1c values were obtained using high-performance liquid chromatography (HPLC) in 54.6 % and turbidimetry and in 40 % of cases; in the remaining patients we used other methods. Levels of fasting and postprandial glycemia, total cholesterol, HDL cholesterol and triglycerides were measured by enzymatic techniques. LDL cholesterol level was calculated using the Friedewald’s equation. The American Diabetes Association’s (ADA) goals for adequate metabolic and clinical control were adopted by the BrazDiab1SG.

Within 1 year of the study assessment, people with a diabetes duration greater than or equal to 5 years from diagnosis were screened for the following chronic diabetes-related complications: microvascular diseases (classified as retinopathy, clinical nephropathy and peripheral neuropathy) and macrovascular diseases (classified as clinical coronary artery disease, stroke, peripheral vascular disease and foot pathologies).

Each center’s local ethics committee approved the study. Written informed consent was obtained from all of the patients or their parents.

### Health-related quality of life assessment

HRQoL was assessed by the EuroQol [11], which includes two tools: the EQ-5D and the EQ-VAS. The first one analyzes descriptively five dimensions of problems (mobility, self-care, usual activities, pain and discomfort, and anxiety and depression) on a scale of three scores, graded from 1 to 3 (1 “I have no problems”, 2 “I have some problems”, 3 “I have extreme problems”). The EQ-VAS (overall health status) consists of an analog scale from 0 (very bad state of health) to 100 (optimal health status), for the patient to check or tell a value that reflects their perception of state of health. The EuroQol is not reliable in individuals younger than 10 years old. Therefore, these participants were excluded from the initial sample. The Portuguese version of the EQ-5D questionnaire has been proven accessible, reliable and valid in measuring health status in a study conducted in Portugal [12]. Additionally, in Brazil, Pinto et al. [13] demonstrated the reproducibility and validity of EuroQol on stroke patients.

Data collected using EQ-5D can be presented in various ways: presenting results from the descriptive system as a health profile, presenting results of the EQ-VAS as a measure of overall self-rated health status, and presenting results from the EQ-5D index value. In order to obtain the EQ-5D index value, it is required a general population-based value set (as opposed to a patient-based set). The rationale behind this is that the values are supposed to reflect the preferences of local taxpayers and potential receivers of healthcare. However, in Brazil, there is not a population-based index available yet. Information in the single index format is useful, for example, in cost utility analysis, which is not the purpose of the present study.

### Statistical analysis

The study sample represented the distribution of T1DM cases across four geographical regions in Brazil, estimated using the overall population distribution reported in the IBGE’s population census of 2000 [14]. These data were combined with the national estimates of diabetes prevalence, which were derived from a 1988 survey, to determine the minimum number of patients to be studied in each region [15]. 3457 patients would be necessary to identify a proportion of patients with adequate glycemic control of 10 %, with a 95 % confidence and a margin of error of 1 %. This number was rounded to 3500 patients and distributed among the four geographical regions [North-Northeast: 1259 (35.7 %); Southeast: 1492 (42.6 %); South: 518 (14.8 %); and Midwest: 240 (6.9 %) individuals]. A total of 3591 patients were included in this study. Participants younger than 10 years old were excluded from the initial sample of 3591 individuals, so that the final sample comprised 3005 patients [North-Northeast: 925 (30.8 %); Southeast: 1180 (39.3 %); South: 718 (23.9 %); and Midwest: 182 (6.1 %)].

Categorical variables were presented as frequency (percentage). All normally distributed values were given as mean ± standard deviation (SD) and all other values were given as median (range). We used Chi squared and Fisher tests to compare categorical data, and T-student and Man-Whitney tests for comparisons between two groups with numeric variables. To test the differences among more than two groups, analysis of variance (ANOVA) was performed for all normally distributed variables, and Kruskal–Wallis test was used for the non-normally distributed variables. For multiple comparisons, the Tukey test (post hoc) was used. For correlation analysis, Pearson or Spearman tests were used.

A two-sided p < 0.05 was considered statistically significant. All data was stored and processed by EPIINFO 2000 and analyzed using the Statistical Package for the Social Sciences version 21.0 (IBM, Chicago, IL, USA) and Sigma Stat version 3.5 (Jandel Scientific Corporation, Chicago, IL, USA).

## Results

The general demographic, clinical and laboratorial data of the assessed population are shown in Table 1.

**Table 1:** General demographic and clinical data of people with T1DM in Brazil

Variables	
Participants, n	3005
Age, years ± SD	23.9 ± 10.8
Women, n (%)	1700 (56)
Age at diagnosis of T1DM in years, n (%)	
0–4.9	330 (11)
5–9.9	721 (24)
10–14.9	932 (31)
15–19.9	492 (16.4)
20–29.9	414 (13.5)
≥30	126 (4.1)
Mean age at diagnosis of T1DM, years ± SD	13 ± 7.9
Duration of T1DM, years	10.9 (7 months–50 years)
Ethnicity, n (%)	
Caucasian	1720 (57.2)
Non-Caucasian	1285 (42.8)
Economic class, %	
High	7.7
Medium	24.3
Low	34.2
Very low	66.1
Geographical region, n (%)	
Southeast	1180 (39.3)
North-Northeast	925 (30.8)
South	718 (23.9)
Midwest	182 (6.0)
Monthly family income, number of minimum wages ± SD	2.3 ± 1.4
Practice of physical activity, n (%)	1723 (57 %)
Smokers, n (%)	150 (5 %)
Microvascular complications, n (%)	673 (29.6)
Macrovascular complications, n (%)	124 (5.5)
Systolic blood pressure, mmHg ± SD	113 ± 16
Diastolic blood pressure, mmHg ± SD	73 ± 11
BMI, kg/m^2^	22.6 ± 3.9
HbA1c, % (mmol/mol)	9.4 ± 2.4 (79 ± 3)
Fasting plasma glucose, mmol/l	10.2 ± 5.8
Total cholesterol, mmol/l	4.4 ± 1.1
Triglycerides, mmol/l	1.1 ± 0.8
LDL cholesterol, mmol/l	2.6 ± 2.1
HDL cholesterol, mmol/l	1.4 ± 0.4
T1DM treatment, %	
Intermediate-acting insulin	16.3
Long-acting insulin	1.3
Insulin pump	1.5
Intermediate- or long- and short-acting insulin	80.8

The average score assigned to general health (EQ-VAS) by people with Type 1 DM in Brazil was of 72.5 ± 22. Analyzing the EuroQol results in each geographical region separately, we have found that the North-Northeast region presents a higher index in the assessment of the overall health status compared to the Southeast. Additionally, the EQ-5D showed a markedly lower frequency of self-reported anxiety-depression in the North-Northeast compared to the other regions of the country (Table 2).

**Table 2:** Euroqol results in T1DM patients of the North-Northeast, Southeast, South and Midwest regions of Brazil

	North-Northeast	Southeast	South	Midwest	p
EQ-VAS score	74.6 ± 30^**┼**^	70.4 ± 19	73.3 ± 17^╞^	71.4 ± 19	<0.05
Mobility	1.06 ± 0.2	1.08 ± 0.3	1.08 ± 0.2	1.1 ± 0.3	NS
Self-care	1.03 ± 0.2	1.03 ± 0.2	1.02 ± 0.2	1.02 ± 0.2	NS
Usual activities	1.08 ± 0.3	1.1 ± 0.3	1.1 ± 0.3	1.15 ± 0.4	NS
Pain and discomfort	1.33 ± 0.5^┬^	1.37 ± 0.6	1.37 ± 0.5	1.48 ± 0.5	<0.05
Anxiety and depression	1.53 ± 0.6^╪^	1.65 ± 0.7	1.72 ± 0.7	1.67 ± 0.7	<0.05

*NS* not significant

^┼^p < 0.05 between North-Northeast versus Southeast

^╞^p < 0.05 between South versus Southeast

^┬^p < 0.05 between North-Northeast versus Midwest

^╪^p < 0.05 between North-Northeast versus Southeast, South and Midwest

The economic status of patients in each geographical region of Brazil is presented on Table 3. We detected that 63.3 % of patients in the Southeast, 56.5 % in the South, 55.5 % in the Midwest and 85.6 % in the North-Northeast region were classified as belonging to low or very low economic class.

**Table 3:** Economic status assessment of T1DM patients in each geographical region of Brazil

Economic class	North-Northeast (%)	Southeast (%)	South (%)	Midwest (%)	p
High	3.8^╪^	8	10.9	12.6	<0.05
Medium	10.6^╪^	28.7	32.6	31.9	<0.05
Low	29.9^╦^	36	37.7	30.2	<0.05
Very low	55.7^╪^	27.3	18. 8^╞^	25.3	<0.05

^╪^p < 0.05 between North-Northeast versus Southeast, South and Midwest

^╦^p < 0.05 between North-Northeast versus South and Southeast

^╞^p < 0.05 between South versus Southeast

Furthermore, we evaluated the clinical and laboratorial variables that could influence the HRQoL of type 1 diabetic patients in the different geographical regions of Brazil (Table 4).

**Table 4:** Clinical and laboratorial variables and their influence on the QoL of T1DM patients in each geographical region of Brazil

Variables	North-Northeast	Southeast	South	Midwest	P
Mean age (years ± SD)	22.5 ± 9^╪^	24.6 ± 11	24.4 ± 11	25.6 ± 10	<0.05
Monthly family income (number of minimum wages ± SD)	2.12 ± 1^╪^	2.49 ± 1.6	2.42 ± 1.3	2.47 ± 1.3	<0.05
Practice of physical activity (n)	499 (54 %)	635 (53 %)	492 (68 %)^╝^	97 (53 %)	NS
Duration of Type 1 DM (years ± SD)	8.6 ± 6.6^╦^	12.2 ± 8.9	11.6 ± 8.4	10.3 ± 7.8	<0.05
HbA1c (% ± SD)	9.6 ± 2.6^┼^	9.2 ± 2.4	9.5 ± 2.1	9.2 ± 2.7	<0.05
Fasting plasma glucose (mg/dl)	185 ± 112	187 ± 109	174 ± 93	171 ± 99	NS
Microvascular complications (%)	22.9^╦^	32.4	32	32.1	<0.05
Macrovascular complications (%)	4^┼^	7.2^╞^	4.3	5.2	<0.05
Micro and macrovascular complications (%)	24.8^╦^	35.5	33.8	32.8	<0.05

*NS* not significant

^╪^p < 0.05 between North-Northeast versus Southeast, South and Midwest

^╦^p < 0.05 between North-Northeast versus South and Southeast

^┼^p < 0.05 between North-Northeast versus Southeast

^╞^p < 0.05 between South versus Southeast

^╝^p < 0.05 between South versus North-Northeast, Southeast and Midwest

On Table 5, we present the correlation between EQ-VAS and the variables that could interfere with HRQoL.

**Table 5:** Correlation between EQ-VAS and the variables that could interfere with HRQoL

Variables	EQ-VAS	
	r	P
Age	−0.1	<0.05
Duration of diabetes	−0.1	<0.05
Practice of physical activity	0.15	<0.05
Economic status	−0.05	<0.05
Monthly family income	0.03	NS
Fasting plasma glucose	−0.1	<0.05
HbA1c	−0.2	<0.05
Microvascular complications	−0.1	<0.05
Macrovascular complications	−0.1	<0.05
Micro and macrovascular complications	−0.1	<0.05

*NS* not significant

## Discussion

This is the first population-based study to evaluate the HRQoL of people with T1DM in the Southern hemisphere, in a country of continental proportions and marked by major regional disparities such as Brazil.

Due to the magnitude of the differences observed among the geographical regions, the pattern that emerges can be defined as markedly asymmetrical. South has, in general, more favorable health indicators than the other regions, as well as a higher degree of homogeneity. The other regions show polarized structures, with North and Northeast presenting predominantly unfavorable indicators, and Southeast and Midwest featuring mostly favorable indicators. A nationwide study on health disparities in Brazil, promoted by the Brazilian Ministry of Health and the Pan-American Health Organization, suggests that three variables seem to explain, in statistical terms, these differences found between the geographical regions, namely: urbanization, poverty and aspects related to the organization of health services. Urbanization suggests the exposure to a “modern” pattern of risk factors. Poverty refers to the difficulties involved in obtaining goods and services directly or indirectly associated to health. Finally, health services can increase or promote health inequalities. In this respect, two variables should be highlighted: access and quality of health services [9].

However, despite these data, the present study has found that the North-Northeast region presents a higher index in the assessment of the overall health status compared to the Southeast, along with a significantly lower frequency of self-reported anxiety-depression compared to all regions of the country. These findings are controversial and cannot be entirely explained by the variables we examined in our study.

The effect of glycemic control on HRQoL of people with T1DM remains unclear, with conflicting studies pointing to opposite directions. A study including teenagers with T1DM suggested that even those who are successfully achieving HbA1c goals of therapy may perceive diabetes as having a negative impact on their lives, be depressed, and find diabetes difficult to manage [16]. In contrast, in a series of over 2000 adolescents with T1DM, Hoey et al. [17] have found that better HbA1c levels were associated with lower impact, fewer worries, greater satisfaction, and better health perception. In the present study, we have found a correlation between HbA1c levels and EQ-VAS. However, our results showed higher levels of HbA1c as well as higher EQ-VAS scores in North-Northeast in comparison to Southeast. Therefore, the better health perception observed in North-Northeast could not be explained by the difference of HbA1c levels. Additionally, the remaining variables that could affect HRQoL individually showed poor correlation with health status.

Regarding the economic status, a cross sectional descriptive study, using a semi structured questionnaire, with 103 diabetic patients, suggested that social class has a significant impact on HRQoL and therapy compliance. The mean scores for general health on upper, middle and lower class were 3.49 ± 0.837, 2.96 ± 0.706, 2.63 ± 0.744 respectively [18]. However, in our study the regions with the highest percentage of people classified as belonging to low or very low economic class, and the lowest monthly family income presented the best HRQoL indicators, which is also paradoxical.

A possible explanation for the better general health status and the lower frequency of self-reported anxiety-depression in North-Northeast is the lower incidence of chronic complications and the shorter duration of diabetes found in this region. These findings are consistent with other studies. Redekop et al. [19] showed that patients with microvascular complications had lower indexes of HRQoL compared to those without chronic complications, even after adjusting for other factors. In addition, other studies suggest that the presence of clinical complications of diabetes have potentially significant impact on HRQoL, since the greater the number of complications, the worse the HRQoL [20]. Furthermore, the BrazDiab1SG, evaluating the HRQoL in Brazil without distinguishing the geographical regions (data not published yet), found weak correlations between the EQ-VAS scores and the micro and macrovascular complications and diabetes duration. Additionally, a linear regression analysis was performed and showed low impact of diabetes complications and diabetes duration on the EQ-VAS score. These data reinforce the hypothesis that there are other factors, not yet evaluated, that could influence the HRQoL.

Regarding the impact of age on HRQoL, a study evaluated patients with type 2 diabetes using the EQ-5D and found that anxiety-depression was more frequently reported by younger patients. A possible explanation for this result was that the projection of future disease progression is more stressful for younger patients. Therefore, the fact that the patients in the North-Northeast region have shown a lower prevalence of anxiety-depression compared to other regions of the country could not be justified by those being younger.

In summary, our results cannot be entirely explained by the variables we examined, such as diabetes duration, chronic complications, HbA1c, age and economic status, suggesting the existence of additional factors not yet evaluated that could be determinant of HRQoL in those individuals.

It has been reported that diabetes mellitus is associated with lower levels of vitamin D [21], which might increase the prevalence of numerous comorbidities, such as depression [22]. In our study, the higher EQ-VAS score and lower frequency of self-reported anxiety-depression in the North-Northeast region of Brazil may perhaps be influenced by vitamin D deficiency. Decreased vitamin D levels tend to be more common in regions of lower sunlight exposure, such as the South and Southeast of Brazil. In a study of 102 non-institutionalized and low-income elderly, in Porto Alegre (Brazilian South region), the prevalence of vitamin D deficiency reached 85.7 % [23]. On the other hand, a study in children in Recife (Brazilian Northeast region) virtually detected no cases of vitamin D deficiency [24].

Other factor possibly related to a poorer HRQoL could be the stressful lifestyle of largely populated cities. The lower frequency of anxiety-depression found in Brazilian North-Northeast, a region with smaller population density, reinforces that hypothesis. The role of lifestyle modification in improving patients’ quality of life, however, is poorly understood. A few studies have shown that lifestyle factors, such as not smoking, getting adequate leisure time physical activity, having a healthy diet and proper sleep and work time, are associated with better HRQoL among people with and without diabetes [25, 26].

The main strength of this study is the recruitment of a large and representative sample of patients with T1DM from each geographical region, in a country of continental proportions such as Brazil, allowing the construction of a national database for a follow-up of these individuals in the long term. Further studies may elucidate the additional variables involved on the HRQoL of these patients, pointing to the need of possible structural, economic and health care-related improvements in each region of the country. We are currently conducting a more specific study on this topic in the North region, which may add, in the future, to the results we have already found. The limitations of this study were: (1) the lack of standardization for the assessment of HbA1c, which could have influenced the results; (2) the self-report nature of doctor-diagnosed diabetes, as there could be misclassification of diabetes, once C-peptide and autoantibodies were not measured.

## Conclusions

In conclusion, this is the first population-based study to assess the HRQoL of people with Type 1 DM in the Southern hemisphere and in a country like Brazil, of continental proportions and marked by major regional disparities. We have found that the North-Northeast region presents a higher index in the assessment of the overall health status compared to the Southeast, along with a significantly lower frequency of self-reported anxiety-depression compared to all regions of the country, which could not be entirely explained by the HbA1c levels or the other variables examined. Thus, our study points to the existence of additional factors not yet evaluated that could be determinant in the quality of life of people with Type 1 DM and contribute to these regional disparities.

## Abbreviations

T1DM: type 1 diabetes mellitus
HRQoL: health-related quality of life
QoL: quality of life
WHOQOL: World Health Organization Quality of Life
DQOL: diabetes quality of life
ADDQoL: audit of diabetes-dependent quality of life
SF-36: Short-Form 36
EQ-5D: EuroQoL 5-dimension
IBGE: Brazilian Institute of Geography and Statistics
BrazDiab1SG: Brazilian Type 1 Diabetes Study Group
NBHCS: National Brazilian Health Care System
BMI: body mass index
HbA1c: glycated hemoglobin
FPG: fasting plasma glucose
LDL: low-density lipoprotein
HDL: high-density lipoprotein
HPLC: high-performance liquid chromatography
ADA: American Diabetes Association
